# Crystal structure and Hirshfeld surface analysis of (*E*)-*N*-[(2-eth­oxy­naphthalen-1-yl)methyl­idene]-5,6,7,8-tetra­hydro­naphthalen-1-amine

**DOI:** 10.1107/S2056989018013117

**Published:** 2018-09-28

**Authors:** Sevgi Kansiz, Mustafa Macit, Necmi Dege, Galyna G. Tsapyuk

**Affiliations:** a Department of Physics, Faculty of Arts and Sciences, Ondokuz Mayıs University, 55139, Samsun, Turkey; b Department of Chemistry, Faculty of Arts and Sciences, Ondokuz Mayıs University, 55139, Samsun, Turkey; c Department of General Chemistry, O. O. Bohomolets National Medical University, Shevchenko Blvd. 13, 01601 Kiev, Ukraine

**Keywords:** crystal structure, Schiff base, Hirshfeld surface

## Abstract

In the title Schiff base compound, C_23_H_23_NO, the two ring systems are twisted by 51.40 (11)° relative to each other. In the crystal, the mol­ecules are connected by weak C—H⋯π inter­actions, generating a three-dimensional supra­molecular structure. Hirshfeld surface analysis and two-dimensional fingerprint plots indicate that the most important contributions to the crystal packing are from H⋯H (67.2%), C⋯H/H⋯C (26.7%) and C⋯C (2.5%) inter­actions.

## Abstract 2

The two ring systems are twisted by 51.40 (11)°. In the crystal, the mol­ecules are linked *via* C—H⋯π inter­actions, forming a three-dimensional framework.

## Chemical context   

Schiff bases have found wide use as a ligands in coordination chemistry (Calligaris *et al.*, 1972 ▸; Hökelek *et al.*, 2004 ▸; Moroz *et al.*, 2012 ▸) and are also important in various areas of chemistry and biochemistry because of their biological activity (El-masry *et al.*, 2000 ▸). Many Schiff bases have some anti­bacterial, anti­cancer and anti­oxidant properties and have therefore been used as starting materials in the synthesis of important medicinal substances. In the present study, we designed a new type of Schiff base obtained by the reaction of 2-eth­oxy-1-naphthaldehyde and 5,6,7,8-tetra­hydro-1-naphtyl­amine to give (*E*)-*N*-[(2-eth­oxy­naphthalen-1-yl)methyl­ene]-5,6,7,8-tetra­hydro­naphthalen-1-amine. We report herein the synthesis, crystal structure and Hirshfeld structural analysis of the title compound.
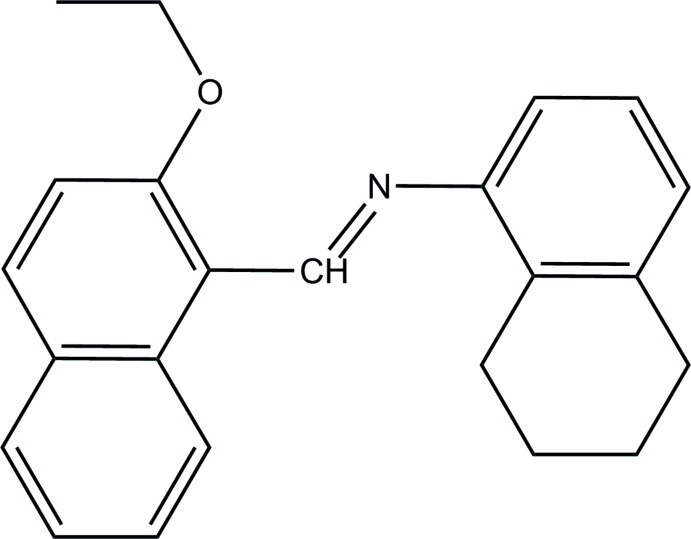


## Structural commentary   

**Figure 1:**
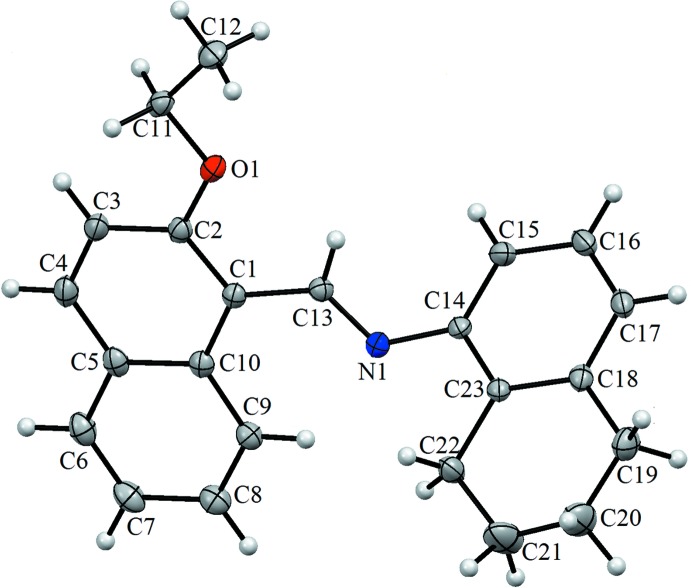
The mol­ecular structure of the title compound, showing the atom labelling. Displacement ellipsoids are drawn at the 20% probability level.

The asymmetric unit of the title compound, (I), contains one independent mol­ecule (Fig. 1 ▸). the two ring systems are twisted by 51.40 (11)° relative to each other. The O1—C2 and O1—C11 bond lengths are 1.359 (4) and 1.423 (4) Å, respectively, while the C13=N1 and C14—N1 bond lengths are 1.262 (3) and 1.415 (5) Å, respectively.

## Supra­molecular features   

**Figure 2:**
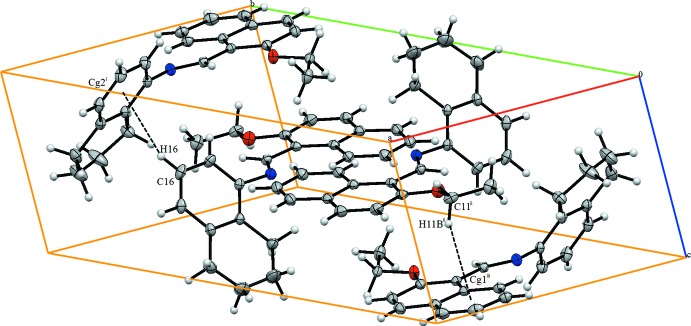
A view of the crystal packing. Dashed lines denote C—H⋯π inter­actions. Symmetry codes: (i) *x*, −*y* + 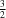, *z* − 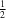; (ii) 1 − *x*, 1 − *y*, 1 − *z*; (iii) 1 − *x*, −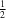 + *y*; 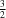 − *z*.

In the crystal, the mol­ecules are connected by C—H⋯π inter­actions, generating a three-dimensional supra­molecular structure (Table 1 ▸ and Fig. 2 ▸).

## Database survey   

There are no direct precedents for the structure of (I) in the crystallographic literature (CSD version 5.39, update of August 2018; Groom *et al.*, 2016 ▸). However, there are several precedents for (*E*)-*N*-benzyl­idene-5,6,7,8-tetra­hydro­naph­thalen-1-amine and (*E*)-*N*-[(2-eth­oxy­naphthalen-1-yl)methyl­ene]aniline including 2-(4-iso­propyl­phen­yl)-1,3-diphenyl-2,3-di­hydro-1*H*-naphtho­[1,2-*e*][1,3]oxazine (Borah *et al.*, 2014 ▸), 2-(2-nitro­phen­yl)-3-(5,6,7,8-tetra­hydro­naphthalen-1-yl)-1,3-thia­zolidin-4-one (Drawanz *et al.*, 2017 ▸), *N*-(3,5-di­meth­oxy­phen­yl)-1,2-di­hydro-3′*H*-spiro­(benzo[*f*]chromene-3,1′-[2]ben­zo­furan)-1-amine (Wu *et al.*, 2013 ▸) and methyl (5a*R*,6a*R*,9*R*,10a*R*)-4-benzoyl-7-methyl4,5,5a,6,6a,7,8,9,10,10adeca­hydro­indolo[4,3-*fg*]quinoline-9-carboxyl­ate dihydrate (Lee *et al.*, 2015 ▸).

## Hirshfeld surface analysis   

**Figure 3:**
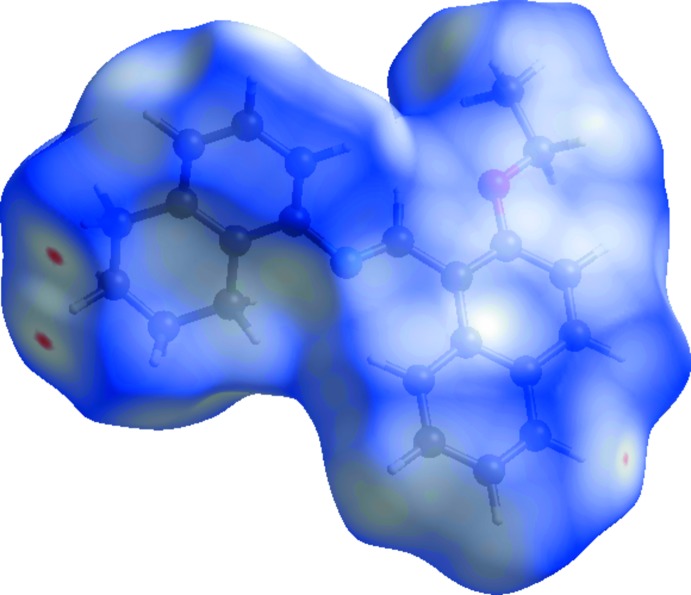
The Hirshfeld surface of the title compound mapped over *d*_norm_.

Hirshfield surface analysis was performed using *CrystalExplorer* (Turner *et al.*, 2017 ▸). The Hirshfeld surfaces and their associated two-dimensional fingerprint plots were used to qu­antify the various inter­molecular inter­actions. The Hirshfeld surface mapped over *d*_norm_ is illustrated in Fig. 3 ▸ [colour scale of −0.067 (red) to 1.262 (blue) Å]. Red spots on this surface indicate the inter­molecular contacts involved in strong hydrogen bonds and inter­atomic contacts (Gümüş *et al.*, 2018 ▸; Kansiz *et al.*, 2018 ▸; Sen *et al.*, 2018 ▸).

**Figure 4:**
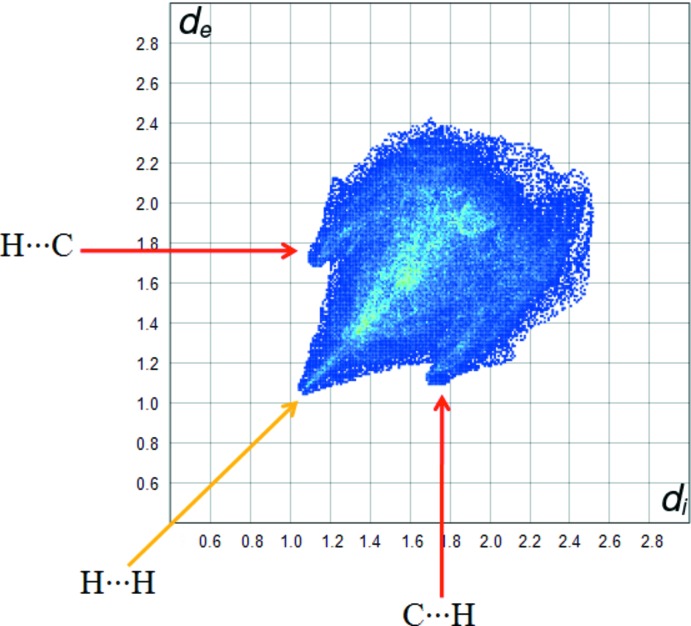
A fingerprint plot for the title compound.

**Figure 5:**
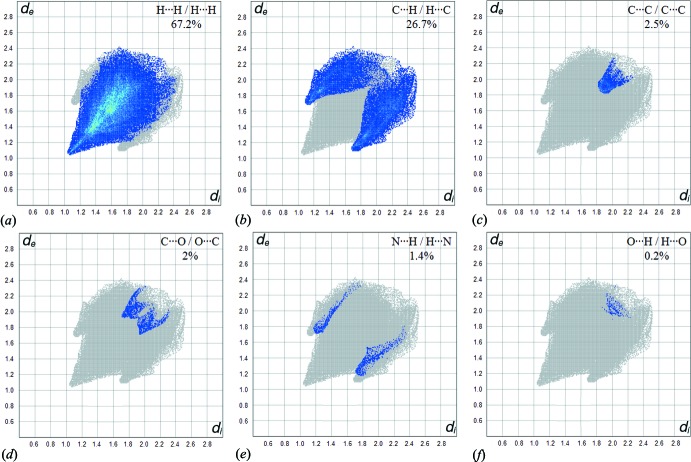
Two-dimensional fingerprint plots for (*a*) H⋯H (67.2%), (*b*) C⋯H/H⋯C (26.7%), (*c*) C⋯C (2.5%), (*d*) C⋯O/O⋯C (2%), (*e*) N⋯H/H⋯N (1.4%) and (*f*) O⋯H/H⋯O (0.2%) contacts.

Fig. 4 ▸ shows the two-dimensional fingerprint of the sum of the contacts contributing to the Hirshfeld surface represented in normal mode. The graph shown in Fig. 5 ▸ *a* (H⋯H) shows the two-dimensional fingerprint of the (*d*_i_, *d*_e_) points associated with hydrogen atoms. It is characterized by an end point that points to the origin and corresponds to *d*_i_ = *d*_e_ = 1.08 Å, which indicates the presence of the H⋯H contacts in this study (67.2%). The graph shown in Fig. 5 ▸ *b* (C⋯H/H⋯C) shows the contacts between the carbon atoms inside the surface and the hydrogen atoms outside the surface and *vice versa*. The plot shows two symmetrical wings on the left and right sides (26.7%). Further, there are C⋯C (2.5%), C⋯O/O⋯C (2%), N⋯H/H⋯N (1.4%) and O⋯H/H⋯O (0.2%) contacts.

**Figure 6:**
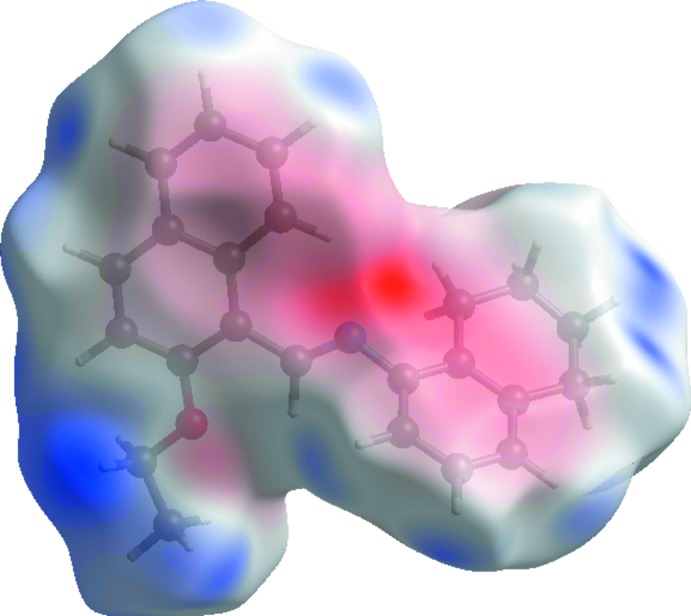
A view of the three-dimensional Hirshfeld surface plotted over electrostatic potential energy.

A view of the three-dimensional Hirshfeld surface of the title compound plotted over electrostatic potential energy in the range −0.048 to 0.033 a.u. using the STO-3G basis set at the Hartree–Fock level of theory is shown in Fig. 6 ▸; the donors and acceptors are shown as blue and red areas around the atoms related with positive (hydrogen-bond donors) and negative (hydrogen-bond acceptors) electrostatic potentials, respectively.

## Synthesis and crystallization   

**Figure 7:**
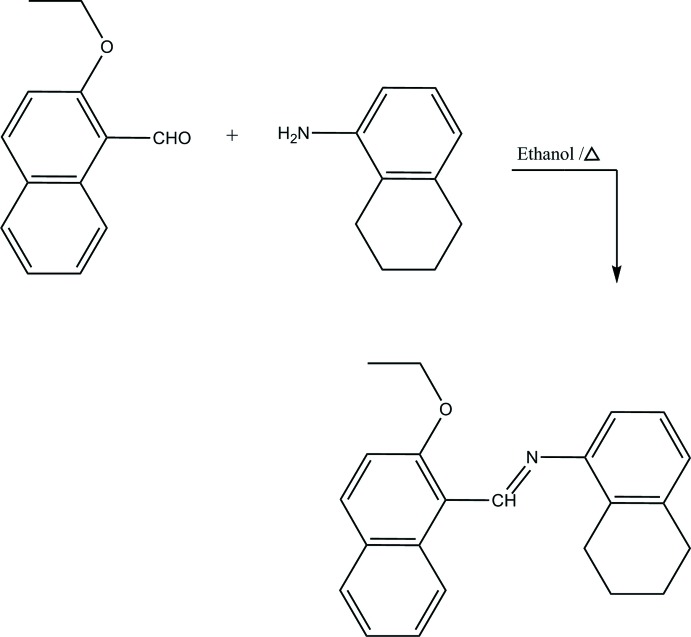
The synthesis of the title compound.

The title compound was prepared (Fig. 7 ▸) by refluxing a mixture of a solution containing 2-eth­oxy-1-naphthaldehyde (20.0 mg, 0.1 mmol) in ethanol (20 mL) and a solution containing 5,6,7,8-tetra­hydro-1-naphtyl­amine (14.72 mg, 0.1 mmol) in ethanol (20 mL). The reaction mixture was stirred for 5 h under reflux. Single crystals of the title compound suitable for X-ray analysis were obtained by slow evaporation of an ethanol solution (yield: 60%; m.p. 416–418 K) .

## Refinement   

Crystal data, data collection and structure refinement details are summarized in Table 2 ▸. Hydrogen atoms were positioned geometrically and refined using a riding model: C—H = 0.93–0.97 Å with *U*_iso_(H) = 1.2*U*_eq_(C).

## Supplementary Material

Crystal structure: contains datablock(s) I. DOI: 10.1107/S2056989018013117/xu5940sup1.cif

Structure factors: contains datablock(s) I. DOI: 10.1107/S2056989018013117/xu5940Isup2.hkl

Supporting information file. DOI: 10.1107/S2056989018013117/xu5940Isup3.cml

CCDC reference: 1843572

Additional supporting information:  crystallographic information; 3D view; checkCIF report

## Figures and Tables

**Table 1:** Hydrogen-bond geometry (Å, °) *Cg*1 and *Cg*2 are the centroids of the C5–C10 and C14–C23 rings.

*D*—H⋯*A*	*D*—H	H⋯*A*	*D*⋯*A*	*D*—H⋯*A*
C11—H11*B*⋯*Cg*1^i^	0.97	2.91	3.799	153
C16—H16⋯*Cg*2^i^	0.93	2.96	3.728	141

Symmetry code: (i) 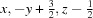.

**Table 2:** Experimental details

Crystal data	
Chemical formula	C_23_H_23_NO
*M*_r_	329.42
Crystal system, space group	Monoclinic, *P*2_1_/*c*
Temperature (K)	296
*a*, *b*, *c* (Å)	12.6628 (4), 20.3304 (9), 7.3838 (3)
β (°)	104.895 (3)
*V* (Å^3^)	1837.01 (13)
*Z*	4
Radiation type	Mo *K*α
μ (mm^−1^)	0.07
Crystal size (mm)	0.61 × 0.47 × 0.25

Data collection	
Diffractometer	Stoe *IPDS* 2
Absorption correction	Integration
*T*_min_, *T*_max_	0.963, 0.982
No. of measured, independent and observed [*I* > 2σ(*I*)] reflections	22781, 3419, 2128
*R*_int_	0.106
(sin θ/λ)_max_ (Å^−1^)	0.606

Refinement	
*R*[*F* ^2^ > 2σ(*F* ^2^)], *wR*(*F* ^2^), *S*	0.081, 0.255, 1.04
No. of reflections	3419
No. of parameters	226
No. of restraints	19
H-atom treatment	H-atom parameters constrained
Δρ_max_, Δρ_min_ (e Å^−3^)	0.44, −0.50

Computer programs: *X-AREA* and *X-RED32* (Stoe & Cie, 2002 ▸), *SHELXL2017/1* (Sheldrick, 2015 ▸), *ORTEP-3 for Windows* and *WinGX* (Farrugia, 2012 ▸) and *PLATON* (Spek, 2009 ▸).

## supplementary crystallographic information

### Crystal data

**Table:**

C_23_H_23_NO	*F*(000) = 704
*M**_r_* = 329.42	*D*_x_ = 1.191 Mg m^−^^3^
Monoclinic, *P*2_1_/*c*	Mo *K*α radiation, λ = 0.71073 Å
*a* = 12.6628 (4) Å	Cell parameters from 16587 reflections
*b* = 20.3304 (9) Å	θ = 1.7–27.9°
*c* = 7.3838 (3) Å	µ = 0.07 mm^−^^1^
β = 104.895 (3)°	*T* = 296 K
*V* = 1837.01 (13) Å^3^	Prism, colourless
*Z* = 4	0.61 × 0.47 × 0.25 mm

### Data collection

**Table:**

Stoe IPDS 2 diffractometer	3419 independent reflections
Radiation source: sealed X-ray tube, 12 x 0.4 mm long-fine focus	2128 reflections with *I* > 2σ(*I*)
Detector resolution: 6.67 pixels mm^-1^	*R*_int_ = 0.106
rotation method scans	θ_max_ = 25.5°, θ_min_ = 1.7°
Absorption correction: integration	*h* = −15→15
*T*_min_ = 0.963, *T*_max_ = 0.982	*k* = −24→24
22781 measured reflections	*l* = −8→8

### Refinement

**Table:**

Refinement on *F*^2^	19 restraints
Least-squares matrix: full	Hydrogen site location: inferred from neighbouring sites
*R*[*F*^2^ > 2σ(*F*^2^)] = 0.081	H-atom parameters constrained
*wR*(*F*^2^) = 0.255	*w* = 1/[σ^2^(*F*_o_^2^) + (0.1536*P*)^2^ + 0.1088*P*] where *P* = (*F*_o_^2^ + 2*F*_c_^2^)/3
*S* = 1.04	(Δ/σ)_max_ < 0.001
3419 reflections	Δρ_max_ = 0.44 e Å^−^^3^
226 parameters	Δρ_min_ = −0.50 e Å^−^^3^

### Special details

**Table:**

Geometry. All esds (except the esd in the dihedral angle between two l.s. planes) are estimated using the full covariance matrix. The cell esds are taken into account individually in the estimation of esds in distances, angles and torsion angles; correlations between esds in cell parameters are only used when they are defined by crystal symmetry. An approximate (isotropic) treatment of cell esds is used for estimating esds involving l.s. planes.

### Fractional atomic coordinates and isotropic or equivalent isotropic displacement parameters (Å^2^)

**Table:**

	*x*	*y*	*z*	*U*_iso_*/*U*_eq_	
C1	0.3883 (2)	0.69673 (14)	0.5168 (4)	0.0625 (7)	
C2	0.3335 (2)	0.75663 (16)	0.4960 (4)	0.0705 (8)	
C3	0.2195 (3)	0.7603 (2)	0.4580 (5)	0.0854 (10)	
H3	0.184711	0.800998	0.441177	0.102*	
C4	0.1603 (3)	0.7044 (2)	0.4460 (5)	0.0881 (11)	
H4	0.084543	0.707238	0.418648	0.106*	
C5	0.2102 (3)	0.64177 (19)	0.4739 (4)	0.0768 (9)	
C6	0.1485 (3)	0.5844 (2)	0.4686 (5)	0.0945 (11)	
H6	0.073038	0.587938	0.447096	0.113*	
C7	0.1946 (4)	0.5244 (2)	0.4937 (5)	0.1018 (12)	
H7	0.151649	0.487132	0.490683	0.122*	
C8	0.3094 (3)	0.51870 (19)	0.5248 (5)	0.0917 (10)	
H8	0.342052	0.477395	0.540440	0.110*	
C9	0.3719 (3)	0.57345 (16)	0.5317 (4)	0.0758 (8)	
H9	0.447121	0.568790	0.552983	0.091*	
C10	0.3259 (2)	0.63747 (15)	0.5075 (4)	0.0666 (8)	
C11	0.3496 (3)	0.87489 (16)	0.5162 (5)	0.0818 (10)	
H11A	0.307563	0.876744	0.608818	0.098*	
H11B	0.301597	0.884936	0.394231	0.098*	
C12	0.4418 (4)	0.92253 (19)	0.5636 (7)	0.1034 (12)	
H12A	0.413575	0.966242	0.566169	0.155*	
H12B	0.488685	0.911972	0.684440	0.155*	
H12C	0.482748	0.920123	0.470930	0.155*	
C13	0.5070 (2)	0.69926 (14)	0.5483 (4)	0.0637 (7)	
H13	0.537591	0.740457	0.541243	0.076*	
C14	0.6849 (2)	0.66195 (13)	0.6046 (4)	0.0620 (7)	
C15	0.7241 (3)	0.69702 (15)	0.4745 (5)	0.0827 (10)	
H15	0.675637	0.716373	0.372129	0.099*	
C16	0.8353 (3)	0.70314 (18)	0.4975 (6)	0.0942 (12)	
H16	0.861669	0.726596	0.410256	0.113*	
C17	0.9070 (3)	0.67484 (17)	0.6482 (6)	0.0859 (10)	
H17	0.981742	0.679759	0.663076	0.103*	
C18	0.8696 (2)	0.63895 (14)	0.7789 (5)	0.0725 (8)	
C19	0.9511 (3)	0.6076 (2)	0.9406 (7)	0.1064 (12)	
H19A	0.998028	0.578883	0.890880	0.128*	
H19B	0.996683	0.641912	1.011812	0.128*	
C20	0.9048 (4)	0.5702 (3)	1.0659 (9)	0.1519 (18)	
H20A	0.944397	0.528870	1.087031	0.182*	
H20B	0.922970	0.593431	1.184568	0.182*	
C21	0.7955 (4)	0.5545 (4)	1.0294 (9)	0.169 (2)	
H21A	0.775939	0.555005	1.148089	0.203*	
H21B	0.787436	0.509438	0.984530	0.203*	
C22	0.7136 (3)	0.5943 (2)	0.8964 (5)	0.0927 (11)	
H22A	0.680842	0.624872	0.966960	0.111*	
H22B	0.656127	0.565356	0.828032	0.111*	
C23	0.7575 (2)	0.63267 (13)	0.7572 (4)	0.0628 (7)	
N1	0.5720 (2)	0.65120 (12)	0.5839 (4)	0.0702 (7)	
O1	0.39688 (19)	0.81145 (11)	0.5147 (4)	0.0871 (7)	

### Atomic displacement parameters (Å^2^)

**Table:**

	*U*^11^	*U*^22^	*U*^33^	*U*^12^	*U*^13^	*U*^23^
C1	0.0606 (16)	0.0751 (19)	0.0516 (14)	0.0063 (14)	0.0141 (12)	−0.0002 (12)
C2	0.0663 (18)	0.083 (2)	0.0631 (16)	0.0111 (15)	0.0186 (13)	0.0067 (14)
C3	0.075 (2)	0.099 (3)	0.087 (2)	0.0223 (19)	0.0294 (17)	0.0176 (18)
C4	0.0625 (19)	0.124 (3)	0.079 (2)	0.012 (2)	0.0196 (15)	0.0103 (19)
C5	0.0659 (18)	0.109 (3)	0.0561 (16)	−0.0054 (18)	0.0159 (13)	0.0011 (15)
C6	0.074 (2)	0.125 (3)	0.083 (2)	−0.018 (2)	0.0178 (17)	−0.009 (2)
C7	0.103 (3)	0.110 (3)	0.093 (3)	−0.039 (3)	0.027 (2)	−0.014 (2)
C8	0.098 (3)	0.090 (2)	0.087 (2)	−0.013 (2)	0.0226 (18)	−0.0086 (18)
C9	0.0753 (19)	0.079 (2)	0.0730 (18)	−0.0078 (16)	0.0182 (14)	−0.0048 (15)
C10	0.0665 (18)	0.083 (2)	0.0504 (14)	0.0030 (14)	0.0149 (12)	−0.0019 (13)
C11	0.102 (2)	0.080 (2)	0.0724 (18)	0.0309 (19)	0.0378 (17)	0.0109 (16)
C12	0.117 (3)	0.075 (2)	0.129 (3)	0.010 (2)	0.050 (3)	−0.003 (2)
C13	0.0644 (16)	0.0644 (17)	0.0626 (16)	0.0029 (14)	0.0169 (13)	0.0032 (12)
C14	0.0608 (16)	0.0511 (14)	0.0773 (17)	0.0031 (12)	0.0236 (13)	0.0022 (13)
C15	0.093 (2)	0.072 (2)	0.091 (2)	0.0124 (17)	0.0383 (18)	0.0218 (16)
C16	0.100 (3)	0.082 (2)	0.123 (3)	0.000 (2)	0.068 (2)	0.014 (2)
C17	0.0700 (19)	0.075 (2)	0.124 (3)	−0.0022 (17)	0.046 (2)	−0.005 (2)
C18	0.0618 (17)	0.0638 (17)	0.093 (2)	−0.0009 (14)	0.0229 (15)	−0.0081 (15)
C19	0.066 (2)	0.119 (3)	0.123 (3)	0.006 (2)	0.0051 (19)	0.007 (2)
C20	0.104 (3)	0.190 (4)	0.142 (4)	0.011 (3)	−0.003 (3)	0.065 (3)
C21	0.121 (3)	0.221 (4)	0.144 (3)	−0.013 (3)	−0.002 (3)	0.097 (3)
C22	0.074 (2)	0.115 (3)	0.090 (2)	−0.0050 (19)	0.0225 (17)	0.031 (2)
C23	0.0603 (16)	0.0541 (15)	0.0766 (18)	−0.0005 (12)	0.0225 (13)	0.0002 (13)
N1	0.0605 (14)	0.0681 (15)	0.0816 (16)	0.0047 (12)	0.0173 (11)	0.0063 (12)
O1	0.0777 (14)	0.0726 (14)	0.1156 (19)	0.0173 (11)	0.0335 (13)	0.0041 (12)

### Geometric parameters (Å, º)

**Table:**

C1—C2	1.390 (4)	C13—N1	1.262 (3)
C1—C10	1.432 (4)	C13—H13	0.9300
C1—C13	1.462 (4)	C14—C15	1.387 (4)
C2—O1	1.359 (4)	C14—C23	1.392 (4)
C2—C3	1.400 (4)	C14—N1	1.415 (4)
C3—C4	1.352 (5)	C15—C16	1.380 (5)
C3—H3	0.9300	C15—H15	0.9300
C4—C5	1.413 (5)	C16—C17	1.368 (5)
C4—H4	0.9300	C16—H16	0.9300
C5—C6	1.398 (5)	C17—C18	1.387 (5)
C5—C10	1.425 (4)	C17—H17	0.9300
C6—C7	1.345 (6)	C18—C23	1.393 (4)
C6—H6	0.9300	C18—C19	1.504 (5)
C7—C8	1.417 (6)	C19—C20	1.434 (7)
C7—H7	0.9300	C19—H19A	0.9700
C8—C9	1.359 (5)	C19—H19B	0.9700
C8—H8	0.9300	C20—C21	1.378 (6)
C9—C10	1.418 (4)	C20—H20A	0.9700
C9—H9	0.9300	C20—H20B	0.9700
C11—O1	1.423 (4)	C21—C22	1.474 (6)
C11—C12	1.488 (5)	C21—H21A	0.9700
C11—H11A	0.9700	C21—H21B	0.9700
C11—H11B	0.9700	C22—C23	1.506 (4)
C12—H12A	0.9600	C22—H22A	0.9700
C12—H12B	0.9600	C22—H22B	0.9700
C12—H12C	0.9600		
			
C2—C1—C10	118.6 (3)	C15—C14—C23	120.1 (3)
C2—C1—C13	116.7 (3)	C15—C14—N1	122.4 (3)
C10—C1—C13	124.7 (2)	C23—C14—N1	117.4 (2)
O1—C2—C1	116.2 (3)	C16—C15—C14	119.8 (3)
O1—C2—C3	121.8 (3)	C16—C15—H15	120.1
C1—C2—C3	121.9 (3)	C14—C15—H15	120.1
C4—C3—C2	119.6 (3)	C17—C16—C15	120.3 (3)
C4—C3—H3	120.2	C17—C16—H16	119.8
C2—C3—H3	120.2	C15—C16—H16	119.8
C3—C4—C5	121.9 (3)	C16—C17—C18	120.9 (3)
C3—C4—H4	119.0	C16—C17—H17	119.6
C5—C4—H4	119.0	C18—C17—H17	119.6
C6—C5—C4	121.4 (3)	C17—C18—C23	119.2 (3)
C6—C5—C10	119.8 (3)	C17—C18—C19	119.2 (3)
C4—C5—C10	118.8 (3)	C23—C18—C19	121.5 (3)
C7—C6—C5	122.2 (4)	C20—C19—C18	115.2 (3)
C7—C6—H6	118.9	C20—C19—H19A	108.5
C5—C6—H6	118.9	C18—C19—H19A	108.5
C6—C7—C8	119.2 (4)	C20—C19—H19B	108.5
C6—C7—H7	120.4	C18—C19—H19B	108.5
C8—C7—H7	120.4	H19A—C19—H19B	107.5
C9—C8—C7	120.1 (4)	C21—C20—C19	123.6 (4)
C9—C8—H8	119.9	C21—C20—H20A	106.4
C7—C8—H8	119.9	C19—C20—H20A	106.4
C8—C9—C10	122.1 (3)	C21—C20—H20B	106.4
C8—C9—H9	119.0	C19—C20—H20B	106.4
C10—C9—H9	119.0	H20A—C20—H20B	106.5
C9—C10—C5	116.6 (3)	C20—C21—C22	120.2 (5)
C9—C10—C1	124.3 (3)	C20—C21—H21A	107.3
C5—C10—C1	119.1 (3)	C22—C21—H21A	107.3
O1—C11—C12	106.6 (3)	C20—C21—H21B	107.3
O1—C11—H11A	110.4	C22—C21—H21B	107.3
C12—C11—H11A	110.4	H21A—C21—H21B	106.9
O1—C11—H11B	110.4	C21—C22—C23	114.8 (3)
C12—C11—H11B	110.4	C21—C22—H22A	108.6
H11A—C11—H11B	108.6	C23—C22—H22A	108.6
C11—C12—H12A	109.5	C21—C22—H22B	108.6
C11—C12—H12B	109.5	C23—C22—H22B	108.6
H12A—C12—H12B	109.5	H22A—C22—H22B	107.6
C11—C12—H12C	109.5	C14—C23—C18	119.7 (3)
H12A—C12—H12C	109.5	C14—C23—C22	119.4 (3)
H12B—C12—H12C	109.5	C18—C23—C22	120.9 (3)
N1—C13—C1	126.6 (3)	C13—N1—C14	119.4 (2)
N1—C13—H13	116.7	C2—O1—C11	120.4 (3)
C1—C13—H13	116.7		
			
C10—C1—C2—O1	177.0 (2)	N1—C14—C15—C16	−176.8 (3)
C13—C1—C2—O1	−2.6 (4)	C14—C15—C16—C17	−0.1 (5)
C10—C1—C2—C3	−2.9 (4)	C15—C16—C17—C18	0.8 (6)
C13—C1—C2—C3	177.5 (3)	C16—C17—C18—C23	−1.0 (5)
O1—C2—C3—C4	−178.1 (3)	C16—C17—C18—C19	178.9 (3)
C1—C2—C3—C4	1.8 (5)	C17—C18—C19—C20	−178.6 (5)
C2—C3—C4—C5	1.1 (5)	C23—C18—C19—C20	1.3 (6)
C3—C4—C5—C6	177.4 (3)	C18—C19—C20—C21	9.9 (10)
C3—C4—C5—C10	−2.8 (5)	C19—C20—C21—C22	−22.6 (12)
C4—C5—C6—C7	179.6 (3)	C20—C21—C22—C23	22.4 (9)
C10—C5—C6—C7	−0.3 (5)	C15—C14—C23—C18	0.2 (4)
C5—C6—C7—C8	−0.6 (6)	N1—C14—C23—C18	176.8 (3)
C6—C7—C8—C9	0.9 (6)	C15—C14—C23—C22	−179.5 (3)
C7—C8—C9—C10	−0.4 (5)	N1—C14—C23—C22	−2.9 (4)
C8—C9—C10—C5	−0.5 (4)	C17—C18—C23—C14	0.5 (4)
C8—C9—C10—C1	178.9 (3)	C19—C18—C23—C14	−179.4 (3)
C6—C5—C10—C9	0.8 (4)	C17—C18—C23—C22	−179.8 (3)
C4—C5—C10—C9	−179.0 (3)	C19—C18—C23—C22	0.3 (5)
C6—C5—C10—C1	−178.6 (3)	C21—C22—C23—C14	168.1 (4)
C4—C5—C10—C1	1.6 (4)	C21—C22—C23—C18	−11.6 (6)
C2—C1—C10—C9	−178.2 (3)	C1—C13—N1—C14	177.9 (3)
C13—C1—C10—C9	1.4 (4)	C15—C14—N1—C13	−49.8 (4)
C2—C1—C10—C5	1.2 (4)	C23—C14—N1—C13	133.7 (3)
C13—C1—C10—C5	−179.3 (3)	C1—C2—O1—C11	−173.1 (3)
C2—C1—C13—N1	174.5 (3)	C3—C2—O1—C11	6.8 (4)
C10—C1—C13—N1	−5.1 (5)	C12—C11—O1—C2	172.7 (3)
C23—C14—C15—C16	−0.4 (5)		

### Hydrogen-bond geometry (Å, º)

Cg1 and Cg2 are the centroids of the C5–C10 and C14–C23 rings.

**Table:**

*D*—H···*A*	*D*—H	H···*A*	*D*···*A*	*D*—H···*A*
C11—H11*B*···*Cg*1^i^	0.97	2.91	3.799	153
C16—H16···*Cg*2^i^	0.93	2.96	3.728	141

Symmetry code: (i) *x*, −*y*+3/2, *z*−1/2.
