# Crystal structure and Hirshfeld surface analysis of (*E*)-*N*-[(2-eth­oxy­naphthalen-1-yl)methyl­idene]-5,6,7,8-tetra­hydro­naphthalen-1-amine. Corrigendum

**DOI:** 10.1107/S2056989018016559

**Published:** 2018-11-30

**Authors:** Sevgi Kansiz, Mustafa Macit, Necmi Dege, Galyna G. Tsapyuk

**Affiliations:** a Department of Physics, Faculty of Arts and Sciences, Ondokuz Mayıs University, 55139, Samsun, Turkey; b Department of Chemistry, Faculty of Arts and Sciences, Ondokuz Mayıs University, 55139, Samsun, Turkey; c Taras Shevchenko National University of Kyiv, Department of Chemistry, 64 Vladimirska Str., Kiev 01601, Ukraine

## Abstract

In the paper by Kansiz *et al.* [*Acta Cryst.* (2018), E**74**, 1513–1516], the address of the correspondence author is incorrect.

## Abstract 2

Erratum to *Acta Cryst.* (2018), E**74**, 1513–1516.

In the paper by Kansiz *et al.* (2018 ▸), the address of the correspondence author, Galyna G. Tsapyuk, should be ‘Taras Shevchenko National University of Kyiv, Department of Chemistry, 64 Vladimirska Str., Kiev 01601, Ukraine’, as given above.
